# Osteomyelitis in heterotopic ossification in a patient with congenital gigantism of the leg

**DOI:** 10.5194/jbji-6-141-2021

**Published:** 2021-04-16

**Authors:** Martina Galea Wismayer, Kurstein Sant, Ryan Giordmaina, Martin McNally

**Affiliations:** 1 Department of Trauma and Orthopaedics, Mater Dei Hospital, Msida, Malta; 2 The Oxford Bone Infection Unit, Nuffield Orthopaedic Centre, Oxford University Hospitals, UK

## Abstract

This paper presents the first report of osteomyelitis in heterotopic ossification in a patient with macrodystrophia lipomatosa. Careful review of magnetic resonance imaging allowed correct diagnosis and design of a limited surgical excision. Osteomyelitis should be considered in the differential diagnosis of pain and discharge when heterotopic ossification is present.

## 1 Introduction

Congenital localized gigantism, more recently known as macrodystrophia lipomatosa (ML), is a rare, congenital and nonhereditary cause of localized limb hypertrophy (Prabhu et al., 2019). It is characterized by abnormal overgrowth of mesenchymal structures in a limb, often within a specific sclerotomal distribution (Prabhu et al., 2019). Although there is a possible association with a PIK3CA gene mutation (Rios et al., 2013), the aetiology remains unclear (Prabhu et al., 2019). It is usually unilateral and diagnosed at birth. Clinically, the affected region grows disproportionately larger in length and girth compared to the rest of the extremity and usually stops at skeletal maturity (Prabhu et al., 2019). Pathologically, it is characterized by hyperproliferation of adipose tissue, interlaced with a fibrous network (Prabhu et al., 2019). ML has been classified into two subtypes depending on the pattern of growth (Barsky, 1967): in the progressive type, the affected region grows at a faster rate compared to the rest of the limb, whereas in the static type, which is more common, there is the same rate of growth for the affected and non-affected areas (Prabhu et al., 2019; Barsky, 1967). Patients usually have major cosmetic concerns and can find the large, heavy limb functionally challenging. Function may also be impaired due to early joint degenerative changes or neurovascular compromise (Prabhu et al., 2019).

## 2 Case report

**Figure 1:**
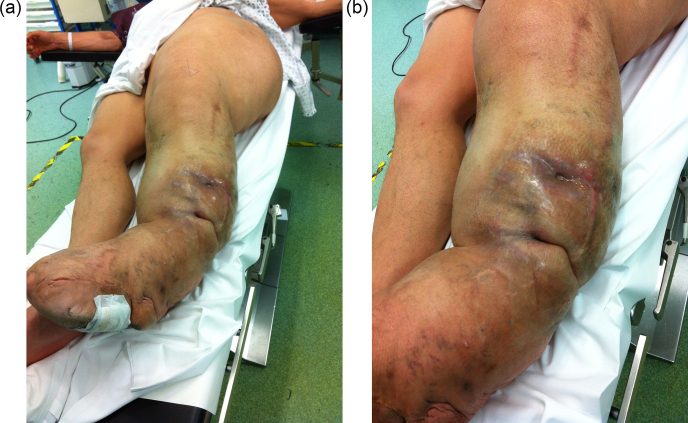
The left leg has the typical features of macrodystrophia lipomatosa, affecting the whole limb and hemipelvis **(a)**. The patient presented with two draining sinuses in an area of indurated skin **(b)**.

A 47-year-old man with gigantism due to static ML of his left leg was referred with a 2-year history of two spontaneously discharging sinuses on the lateral aspect of his left leg (Fig. 1a and b). He had a history of soft tissue debulking 30 years prior to presentation which was complicated by postoperative haematoma formation needing surgical drainage. After this surgery, his leg healed well. There were no concerns about infection at that time and he had no sinus drainage. He returned to his previous mobility and had no problems with the leg for over 20 years.

He recalled sustaining blunt trauma to the front of his left leg a number of years prior to presentation. This was a minor injury but was complicated by a cellulitic reaction in the skin. This persisted for a few weeks and then settled completely. Again, he had no skin breakdown or sinus formation related to this injury.

He was reviewed several times over the following years by his local surgeons, with mobility issues due to the large bulk of his leg. He then presented with pain and new spontaneous sinus formation, without any new injury or other infection. It was assumed that he must have haematogenous osteomyelitis of the tibia or fibula. Treatment options were discussed. It was decided that resection of infected bone from the tibia or fibula would be possible but that closure of the wound in this very large leg would be problematic. Amputation was also considered. He was reviewed by a plastic surgeon who concluded that amputation would not be possible as the gigantism extended to above the hip joint and would require a very major reconstruction to close the residual stump (Fig. 1a). Prosthetic fitting would be challenging. Similarly, local excision would require a free tissue transfer which would be extremely difficult in this huge lower limb. It was therefore considered that this was not operable due to his massive limb enlargement.

**Figure 2:**
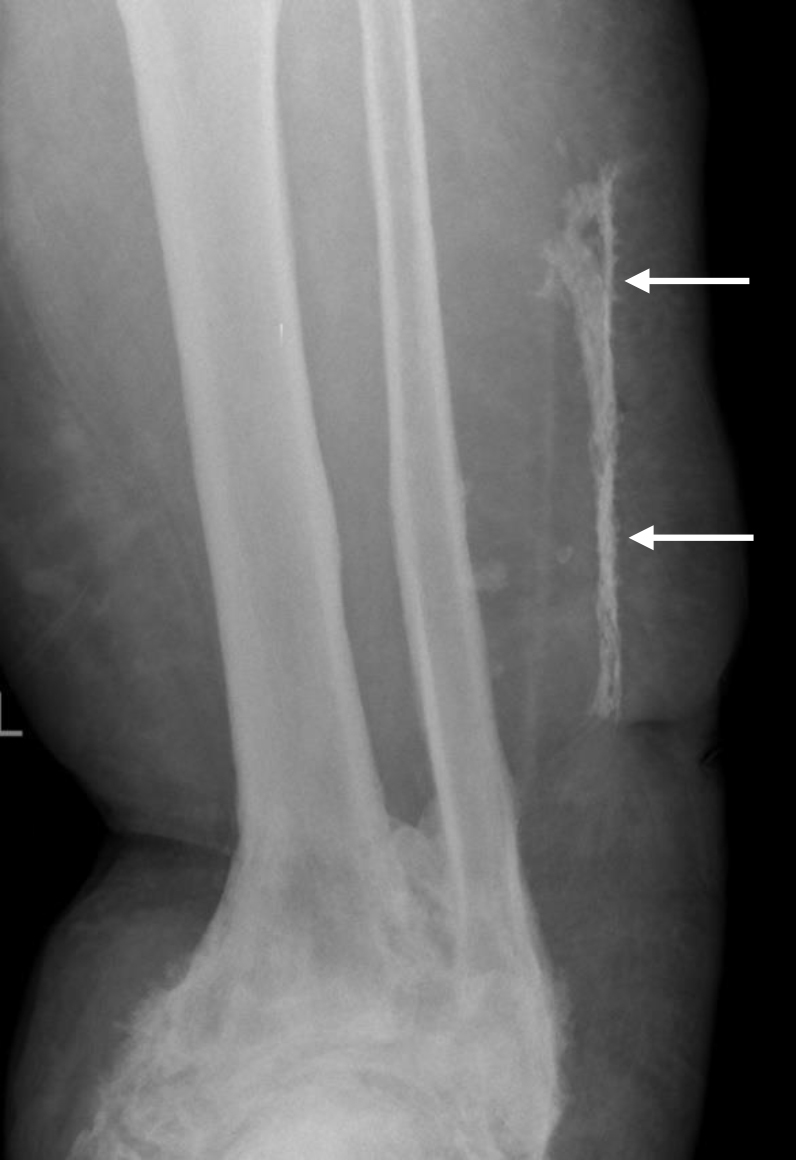
Plain X-ray showed the area of new bone formation in the lateral soft tissues (white arrows), separate from the fibula. The ankle joint has extensive heterotopic bone formation, deformity and ankylosis.

**Figure 3:**
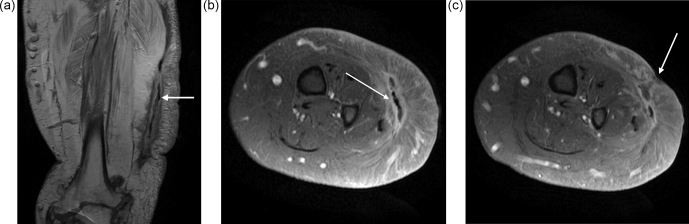
MRI revealed the extent of the infection around the heterotopic bone (white arrow). The muscles under the HO have undergone significant fatty replacement **(a)**. Transverse images **(b)** show the high signal around the HO with oedema in the muscle and subcutaneous tissue (white arrow). There is no evidence of infection around the tibia or fibula and the surrounding muscles are normal. The fluid around the HO extended to a skin sinus (white arrow) **(c)**.

At presentation to us, he was systemically well. The soft tissues on the lateral aspect of his left leg were indurated with two obvious draining sinuses (Fig. 1b). A hard, non-tender lump was palpable deep to his scars and sinuses. There was no tenderness along the tibia and fibula. Plain radiography showed subcutaneous calcification consistent with heterotopic ossification or possible sequestration and infection (Fig. 2). There was no periosteal reaction, lysis or sequestration in the fibula or tibia. A magnetic resonance imaging (MRI) scan demonstrated fluid around the heterotopic bone, with oedema in the surrounding soft tissues (Fig. 3a, b). This fluid extended to the skin at one of the sinuses (Fig. 3c). The tibia and fibula were normal. There was no sign of any tumour or other pathology.

**Figure 4:**
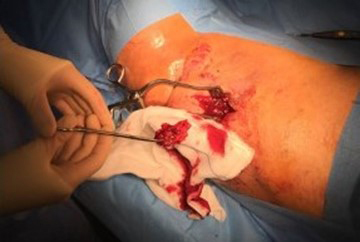
At operation, the infected HO was found deep to the subcutaneous fat, on the surface of the muscle. It separated easily from the tissues and was removed.

**Figure 5:**
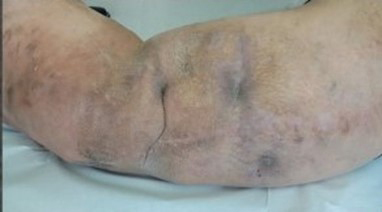
At 1 year after surgery, the surgical site is fully healed with no recurrence of infection.

Intraoperative findings confirmed the presence of subcutaneous heterotopic ossification with osteomyelitis, with no deeper extension of the infection. The ossification had formed superficial to the deep fascia in the lower dermis. At the start of the operation, the sinus tract was excised and discarded. The heterotopic bone was exposed and five samples were taken from this bone and the soft tissue attached to it, with separate instruments. These were cultured for anaerobic and aerobic organisms. The infected and dead bone was excised completely and the tissues were closed primarily (Fig. 4). Intraoperative bone cultures showed *Enterobacter cloacae*, *Pseudomonas aeruginosa* and *Staphylococcus aureus*. All three organisms were cultured from at least three specimens. The *Staph. aureus* was fully sensitive and the Gram negatives were only resistant to nitrofurantoin, Ceftazidime and Aztreonam. He was treated with intravenous amikacin and ciprofloxacin according to these sensitivity results, followed by 6 weeks of oral ciprofloxacin. He recovered quickly from surgery with good wound healing and return to weight-bearing. At 1-year follow-up, the wound over the lateral leg was well healed and there were no signs of local recurrence of osteomyelitis (Fig. 5). The patient has been reviewed at 30 months after surgery and has remained infection-free, with no recurrence of the sinus and no local signs of infection.

## 3 Discussion

Localized limb hypertrophy may be caused by a number of conditions, including ML, neurofibromatosis type 1, Klippel–Trenaunay–Weber syndrome, haemangiomatosis and Proteus syndrome (Khan et al., 2010). However, with the exception of ML, these conditions usually have a positive family history and are associated with cutaneous and systemic signs (Khan et al., 2010). Although mostly asymptomatic, patients with ML typically present with two primary concerns: cosmetic and mechanical. Cosmesis may be the presenting complaint at all ages but mechanical problems tend to develop in adolescence with secondary degenerative joint changes and resultant reduction in function (Prabhu et al., 2019; Khan et al., 2010). Patients may also present due to repetitive trauma to the affected region (Khan et al., 2010). This is likely to be due to the sheer size of the affected region, making it more prone to injury.

Heterotopic ossification forms within soft tissues which normally do not ossify (Mujtaba et al., 2019). It can either be genetic or acquired. The latter is more common, often secondary to soft tissue trauma, including burns, orthopaedic procedures, musculoskeletal trauma and brain and spinal cord injury (Mujtaba et al., 2019). Osteomyelitis has not been described as a complication of heterotopic ossification (HO) and it is more common to have HO forming in response to infection, particularly around infected joint prostheses (Ohlmeier et al., 2019).

It is difficult to determine when the heterotopic ossification developed in this limb. It may have developed in the scar after the debulking surgery, particularly as there was a history of haematoma formation. Ossification of limb haematomas was first described by Van Arsdale in 1893 (Van Arsdale, 1893), but there are no reports of osteomyelitis developing in such bone. However, our patient was completely unaware of any HO after this surgery and his infection did not present until many years later. We suspect that his HO was secondary to the blunt trauma sustained to his left leg several years prior to presentation. This trauma produced a small haematoma along the fascia which may have ossified, forming an area of HO. Our patient reports a “cellulitic episode” following his traumatic event. This may have been a true episode of skin cellulitis, but his symptoms of erythema, swelling, pain and increased warmth may alternatively have been due to the initial inflammatory phase of HO formation which can present with these symptoms (Shehab et al., 2002). The two diagnoses are in fact often confused (Meyers et al., 2019). A review of 235 patients with HO after spinal cord injury reported that 6.4 % exhibited features which mimicked active infection (infective symptoms, high CRP, body temperature above 38.5 ${}^{\circ }$C; Citak et al., 2016).

The origin of the osteomyelitis is assumed to be haematogenous, arising around 2 years prior to presentation, as he did not suffer any new injury and had no limb surgery. He did have a small ulcer over the lateral border of his foot which may have seeded infection to the area of HO. The presence of spontaneously draining sinuses, imaging and polymicrobial cultures from multiple deep samples confirmed the diagnosis.

In this case, the diagnosis of HO was confirmed by plain X-ray and MRI. On MRI, typical “striate” and checkerboard-like patterns seen on the T2-weighted images help to distinguish this condition from osteomyelitis and sarcomas (Mujtaba et al., 2019). As the HO matures, with calcification developing at its periphery, findings on MRI become less specific, making diagnosis more difficult. However, once fully formed, the characteristic features of hyperintense cancellous marrow on T1- and T2-weighted images with a rim of hypointense cortical bone make the diagnosis clearer (Mujtaba et al., 2019). Mature, uninfected HO should not exhibit peri-osseus or medullary oedema, which are more characteristic of osteomyelitis (Lee et al., 2016). In this case, the presence of long-standing sinuses diagnosed the osteomyelitis, which was confirmed and localized by the imaging. Newer imaging modalities such as single-photon emission computed tomography in combination with computed tomography (SPECT-CT) are increasingly being used to aid in the diagnosis of HO. SPECT-CT has also been used successfully to distinguish between osteomyelitis and HO (Hassan et al., 2012).

## 4 Learning point

Osteomyelitis has not previously been reported in gigantism or post-traumatic HO in the limbs. Clinical review of this patient suggested a typical lower limb chronic osteomyelitis, arising in the tibia or fibula. The presence of severe gigantism prevented simple surgical resection of this, so he was denied curative treatment. Careful review of the MRI scans revealed no involvement of his tibia or fibula but demonstrated an area of unexpected HO in the soft tissues on the lateral aspect of his leg, communicating with the sinus tracts. This was much more amenable to surgical resection with easy soft tissue closure.

## Data Availability

All available data are presented within the text and figures of this paper.
